# Benchmarking of provider competencies and current training for prevention and management of obesity among family medicine residency programs: a cross-sectional survey

**DOI:** 10.1186/s12875-021-01484-y

**Published:** 2021-06-24

**Authors:** Manuela Orjuela-Grimm, W. Scott Butsch, Silvia Bhatt-Carreño, B. Gabriel Smolarz, Goutham Rao

**Affiliations:** 1 Departments of Epidemiology and Pediatrics, Mailman School of Public Health, Columbia University, New York, NY USA; 2 Departments of Surgery and Internal Medicine and Geriatrics, Bariatric and Metabolic Institute, Cleveland Clinic, Cleveland, OH USA; 3 Novo Nordisk Inc., Plainsboro Township, NJ USA; 4 Department of Family Medicine and Community Health, Case Western Reserve University, Cleveland, OH USA

**Keywords:** Obesity, Education, Internship and residency, Family practice, Graduate medical education, Primary health care

## Abstract

**Background:**

U.S. physicians lack training in caring for patients with obesity. For family medicine, the newly developed Obesity Medicine Education Collaborative (OMEC) competencies provide an opportunity to compare current training with widely accepted standards. We aimed to evaluate the current state of obesity training in family medicine residency programs.

**Methods:**

We conducted a study consisting of a cross-sectional survey of U.S. family medicine residency program leaders. A total of 735 directors (including associate/assistant directors) from 472 family medicine residency programs identified from the American Academy of Family Physicians public directory were invited via postal mail to complete an online survey in 2018.

**Results:**

Seventy-seven program leaders completed surveys (16% response rate). Sixty-four percent of programs offered training on prevention of obesity and 83% provided training on management of patients with obesity; however, 39% of programs surveyed reported not teaching an approach to obesity management that integrates clinical and community systems as partners, or doing so very little. Topics such as behavioral aspects of obesity (52%), physical activity (44%), and nutritional aspects of obesity (36%) were the most widely covered (to a great extent) by residency programs. In contrast, very few programs extensively covered pharmacological treatment of obesity (10%) and weight stigma and discrimination (14%). Most respondents perceived obesity-related training as very important; 65% of the respondents indicated that expanding obesity education was a high or medium priority for their programs. Lack of room in the curriculum and lack of faculty expertise were reported as the greatest barriers to obesity education during residency. Only 21% of the respondents perceived their residents as very prepared to manage patients with obesity at the end of the residency training.

**Conclusion:**

Family medicine residency programs are currently incorporating recommended teaching to address OMEC competencies to a variable degree, with some topic areas moderately well represented and others poorly represented such as pharmacotherapy and weight stigma. Very few program directors report their family medicine residents are adequately prepared to manage patients with obesity at the completion of their training. The OMEC competencies could serve as a basis for systematic obesity training in family medicine residency programs.

**Supplementary Information:**

The online version contains supplementary material available at 10.1186/s12875-021-01484-y.

## Background

Obesity is a serious public health problem in the United States (U.S.) affecting roughly 40% of American adults [1]. Obesity may cause, exacerbate, or accelerate numerous medical and psychosocial conditions including diabetes, heart disease, cancer, and depression and it also has profound economic consequences [2, 3]. The direct costs attributed to the medical complications of obesity have been estimated at $190 billion annually [4]. Indirect costs, including lost productivity, have been estimated to be an additional $1.24 trillion dollars [5]. Unfortunately, rates of obesity continue to climb [1].

Recent clinical guidelines reflect the recognition of a need to integrate obesity management in clinical care, and provide clear recommendations for medical care of patients with obesity [6, 7]. However, management of obesity has not been prioritized [8] or managed effectively in primary care settings [9–11]. In fact, despite high prevalence rates, fewer than 5% of primary care visits in 2008 were dedicated to obesity based on the National Ambulatory Medical Care Survey [12]. In addition to lack of sufficient clinic time and inconsistent reimbursement for obesity related codes [13], other reasons why primary care physicians may not effectively address obesity include discomfort discussing obesity with patients [14] and limited exposure to obesity as part of formal education in both medical school and residency. Studies have shown that less than one-third of medical schools meet the minimum recommended hours of nutrition education [15] and there is limited coverage of obesity-related topics in internal medicine programs [16] or medical licensing exams [17].

Successfully tackling the obesity epidemic requires a multi-faceted approach that includes innovative new treatments, changes in public health policy, improved public awareness of the causes and consequences of obesity, and improved provider education and training. In 2019, the Obesity Medicine Education Collaborative (OMEC) formulated a set of 32 obesity-related competencies for medical students, trainees, and professionals engaged in the diagnosis, evaluation, counseling, and treatment of patients with obesity [18]. This complemented an earlier effort by the Provider Training and Education Workgroup of the Integrated Clinical and Social Systems for the Prevention and Management of Obesity Innovation Collaborative which had created obesity competencies for inter-professional education [19]. These competencies have been endorsed by a number of professional organizations including the American Academy of Family Physicians (AAFP) and the Society of Teachers of Family Medicine (STFM). Family physicians are in an ideal position to care for patients with obesity because obesity is often a lifelong, relapsing condition for which a meaningful long-term relationship with a healthcare provider is appropriate [20].

Our objective was to obtain a baseline assessment documenting current training on the management of obesity incorporated in family medicine programs, which components are included, and the consistency of this training with the recently published OMEC competencies. Ultimately our goal for this research is to serve as a benchmark against which to examine incorporation of the OMEC competencies in future assessments. The aim of this study is to: 1) describe the proportion of family medicine residency programs with training programs for care of patients with obesity, 2) examine whether family medicine residents are adequately prepared to manage patients with obesity at the end of their training, and 3) describe the extent to which the OMEC competences are currently addressed in family medicine residency programs.

## Methods

### Study design

We surveyed leaders of family medicine residency programs in the U.S. between October 23 and December 7, 2018. Invitations were sent by postal mail and surveys were completed online. Although more than one leader in some programs was identified, only one respondent was permitted from each residency program to ensure consistent data and representation across institutions. The study was reviewed by the Columbia University Institutional Review Board and was found to qualify for exempt status (IRB-AAAS0063; 16.8.2018).

### Participants

We identified potential study participants through a multi-step process. Using the AAFP public directory [21], we selected all 474 family medicine residency programs listed. Based on addresses in the AAFP directory of residency programs and after excluding the two institutions from which the only identified contacts participated in the pilot testing, we mailed 735 invitations to program leaders including directors, associate directors, and assistant directors that we could identify using publicly available information including residency program websites. The mailed package included a letter specifying the study sponsor (Novo Nordisk) and academic collaborators (Columbia University Mailman School of Public Health and the Bariatric and Metabolic Institute at the Cleveland Clinic), study objectives, participation requirements, web link and instructions for completing the online survey, and a prepaid incentive of $65 in the form of a check to thank participants for their time. A second postal mailing, along with several rounds of follow-up faxes and emails, were used to remind non-responders to participate. To qualify, respondents were required to be at least somewhat familiar with “the Accreditation Council for Graduate Medical Education’s (ACGME’s) learning objectives and requirements for family medicine” to ensure survey responses were based on sufficient knowledge of their residency program.

### Survey instrument

The online survey was based on the OMEC competencies and comprised 47 questions related to obesity education, including multiple choice, scalar, and numeric text questions. Respondents were asked to assess the degree to which their residency program curricula address core obesity competencies on a 4-point Likert scale (“great extent”, “some extent”, “very little extent”, and “not at all”). Survey questions included the nature and setting of obesity training in the curriculum, opportunities for clinical rotations in obesity, as well as expectations, priorities, and barriers regarding expansion of obesity education.

We based the family medicine survey on a similar survey of medical schools [22] and internal medicine residency programs [16]. To emphasize unique aspects of family medicine, the survey was adapted, through an iterative process, to include questions related to the prevention of obesity (in addition to its management), the extent to which programs integrate clinical and community systems as partners in obesity management, the degree to which patient-centered communication is emphasized, and the level of participation of other disciplines as part of the “medical home” team. See Additional file 1 for the survey questions.

Prior to fielding the survey, a family residency faculty member reviewed and suggested modifications to the instrument. The resulting instrument was then pilot-tested with four family medicine residency program directors via telephone and a web-based platform for their assessment of the face validity of the survey. We made minor wording changes to the survey based on feedback from the pilot to improve clarity and relevance.

### Statistical analysis

We used descriptive statistics to summarize respondents, programs, and responses. Univariate comparisons of responses by key independent variables were completed using Spearman’s rank correlation test. All statistical analyses were completed using SPSS v. 24 (IBM, Armonk, NY). Results were considered statistically significant if the *p*-value < 0.05.

## Results

### Characteristics of respondents and family medicine residency programs

Leaders of 77 programs completed the survey including 56 program directors and 21 other residency leaders (of a total of 472 programs approached; 16% response rate). Respondents completed the survey in a median of 12 min. All respondents were involved in teaching/training residents and had been in their current role for a mean of 7 years. Eighty-eight percent of the programs were associated with medical schools (i.e., medical school-based, -administrated, or -affiliated) and 12% were community-based, non-affiliated programs (Table 1).

**Table 1:** Characteristics of 2018 family medicine residency online survey respondents and their institutions (*n* = 77)

**Title/Role**	**n (%)**^a^	
Program Director	56 (72.7)	
Associate Director or Co-Director	15 (19.5)	
Assistant Director	5 (6.5)	
Other^b^	1 (1.3)	
**Gender**	**n (%)**^a^	
Male	51 (66.2)	
Female	26 (33.8)	
**Academic Experience**	**Mean (mean ± SD)**	
Mean years at current institution	12.7 ± 8.5	
Mean years in current role	6.8 ± 6.4	
**Family Medicine Residency Programs**	**Survey Sample (%)** **(*n* = 77)**	**U.S. Family Medicine Programs (%)** **(*n* = 474)**
**Program Affiliation**^a, c^		
Community-Based, Med School Administered	19.0	16.2
Community-Based, Med School Affiliated	49.0	59.5
Community-Based, Non-Affiliated	12.0	9.7
Med School-Based	19.0	12.0
Military	0	2.5
**Population Served**^c,d^		
Inner-City	6.0	6.8
Urban	60.0	50.0
Suburban	38.0	44.7
Rural	27.0	31.0
**Geographic Region**^a, c^		
Northeast	18.2	16.9
Midwest	31.2	28.5
South	32.5	32.3
West	18.2	22.4

*SD* Standard deviation

^a^Percentages may not sum to 100% due to rounding

^b^One respondent specified “faculty” in the “other” response option

^c^Data is from the AAFP residency database (last accessed in 2018)

^d^Programs might identify as serving more than one population

### Obesity in the family medicine residency curriculum

Most respondents reported that their residency programs offered formal or organized training on obesity topics: 49 (64%) programs on prevention of obesity and 64 (83%) programs on management of patients with obesity. Fifty-two percent of all the surveyed programs offered surgical clinical rotation opportunities and 43% had non-surgical rotations in obesity. In addition to clinical faculty, residents from 69 programs (90% of total programs surveyed) had opportunities to work with other healthcare providers who cared for patients with obesity, although the nature of this work was not defined in the survey nor was the extent to which residents participated in these opportunities. Nearly all (99%) respondents reported that other clinical disciplines participated in medical home teams and inpatient rounds, including nursing (79%), social work (75%), psychology (58%), nutrition (42%), pharmacy (30%), and physical therapy (25%).

Although 49% of programs reported teaching an approach to obesity management that integrates clinical and community systems as partners to “some extent”; only 12% indicated doing so to a “great extent”, 27% “very little”, and 12% reported not teaching this approach at all. Furthermore, only 47% of participants reported that their programs emphasized to a “great extent” the use of patient-centered communication when working with patients with obesity, while 12% indicated their program emphasized this very little or not at all.

**Fig. 1:**
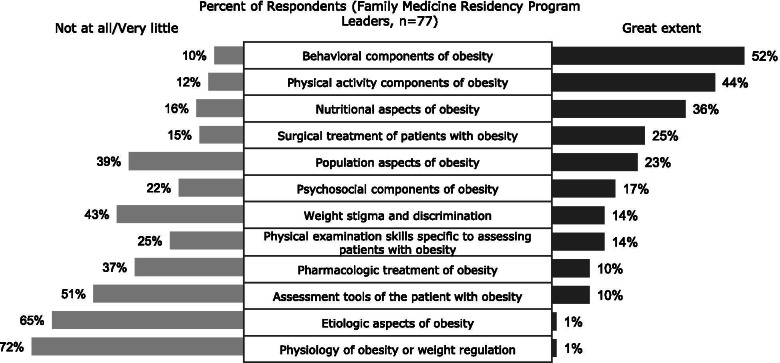
Coverage of Obesity Competencies in Family Medicine Residency Programs. Some competencies have been shortened for presentation; responses of “some extent” not shown

Figure 1 summarizes the extent of coverage of core obesity competencies during the family medicine residency program. There were notable differences in coverage across topic areas. Among the core topics on obesity included in the survey, only behavioral aspects of obesity were covered to a great extent by slightly more than half of the programs. Nutrition aspects of obesity and physical activity were covered to a great extent in 36% and 44% of programs, respectively. The topic of weight stigma and discrimination were covered to a much lesser extent. Teaching was provided primarily in supervised outpatient clinics and dedicated seminars.

Physical exam skills specific to assessing patients with obesity were reported to be covered at least to some extent in the majority of programs; however, additional assessment tools (metabolic testing, body composition analyses) were covered very little or not at all by half of the programs. Teaching of this component was primarily in an outpatient clinic setting.

Treatment of obesity was generally not extensively covered. Surgical treatment was more commonly addressed than was pharmacological treatment. Only 10% of programs reported covering the latter to “great extent” – over one-third reported no or very little coverage. These topics were taught primarily in outpatient precepting clinics and dedicated seminars of respondents’ programs.

Finally, competencies related to the etiology and pathophysiology of obesity were the least addressed in the curriculum, with more than half of the programs covering those topics to a very little extent and at least 10% not covering them at all; both topics were typically taught in dedicated seminars. The extent to which etiologic aspects of obesity were included in the curricula was highly correlated with the extent to which physiologic (hormonal) aspects of obesity were covered (*r* = 0.53; *p* < 0.001).

### Perception of preparedness in managing patients with obesity

**Fig. 2:**
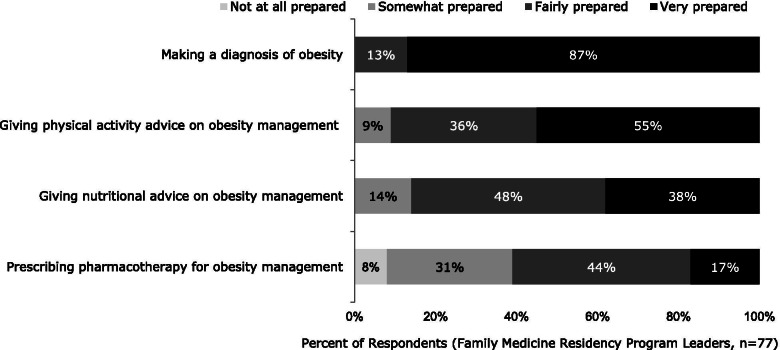
Respondents’ Personal Preparedness for Managing Patients with Obesity

Most respondents (program leaders) (87%) perceived themselves as “very prepared” to diagnose obesity. Approximately 55% and 38% felt that they were “very prepared” to give physical activity advice and nutritional advice for obesity management, respectively (Fig. 2). In contrast, only 17% of respondents described themselves as “very prepared” to prescribe pharmacotherapy for obesity management and 8% felt that they were “not at all prepared”. Interestingly, respondent’s perception of their preparedness for prescribing pharmacotherapy was significantly correlated with the perceived degree of importance of pharmacotherapy in obesity education (*r* = 0.28; *p* < 0.02), as well as to the extent that pharmacotherapy is included in the residency program curriculum (*r* = 0.32; *p* < 0.005).

When asked about the preparation level of their residents at the end of the residency program, only 21% of the respondents felt that the residents were “very prepared” to treat patients with obesity. Slightly more than half (53%) reported that their residents were “fairly prepared”, and the remainder perceived their residents as only “somewhat prepared” (25%) or “not at all prepared” (1%). Resident preparation was significantly correlated with the degree of preparation the respondents (i.e., program directors) had for giving nutritional advice (*r* = 0.46; *p* < 0.001).

### Perceived importance of obesity-related education

**Fig. 3:**
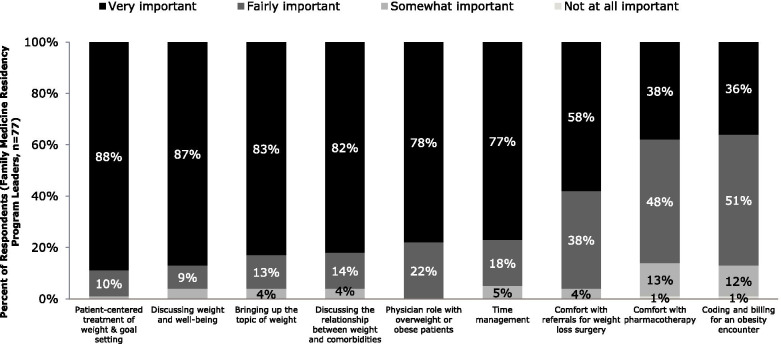
Importance of Obesity-Related Topics for Education

When asked about the importance of education on specific obesity-related topics, most respondents (58%–88%) rated nearly all as “very important” (Fig. 3); the two exceptions were comfort with obesity pharmacotherapy and coding and billing for an obesity encounter, which were rated as “very important” by only roughly one-third of the respondents.

We inquired about incorporating obesity education into the curriculum: three-quarters (75%) of respondents thought it should be offered as both a separate discipline and as part of education related to the management of other related medical conditions; only 4% felt obesity education should be taught solely as a separate discipline and 21% felt it should only be incorporated as portions of teaching in conjunction with management of other conditions. Recognition of the importance of including obesity education in family medicine residency training was consistent with respondents’ estimates of the high mean prevalence of overweight (39%) and obesity (32%) in the patient populations served by their residents.

### Prioritizing obesity education in family medicine residency curriculum

Almost two-thirds of respondents indicated that expanding obesity education was a high (10%) or medium (55%) priority for their programs; of these programs, 34% had plans to update (either incorporate or expand) the obesity curriculum within 1 year and 50% within 1–2 years. The top challenges related to integrating obesity education during residency were lack of room in the curriculum and lack of faculty expertise in obesity; these challenges were perceived as large or moderate barriers by 56% and 44% of the respondents, respectively. In contrast, fewer than 21% of respondents reported lack of faculty or resident interest and financial considerations as significant barriers.

## Discussion

We sought to evaluate the current state of obesity education in family medicine residency program curricula and to gauge its alignment with the OMEC competencies. Our analysis can help identify priorities for future obesity education.

We found that the OMEC competencies are addressed to some extent in U.S. family medicine residency programs, but coverage varies widely by topic, with greater emphasis on areas such as behavioral, physical, and nutritional aspects of obesity and much less attention given to the clinical assessment and treatment of patients with obesity. Additionally, although most programs offered formal training on topics related to prevention and management of obesity, the actual didactic and clinical time devoted specifically to management of patients with obesity was limited. Thus, it is not surprising that only one-fifth of program leaders rated their residents as being very prepared to manage patients with obesity at the end of training. These findings underscore the need for improving obesity-related training during family medicine residency.

The few studies that have examined obesity in residency curricula have focused on internal medicine [23–25] or pediatrics [26]. A survey of Ohio medical residents (family, internal, and obstetrics and gynecology) found low levels of knowledge about obesity and counseling practices as well as low levels of perceived self-efficacy to effectively counsel their patients on obesity, nutrition, and physical activity [27]; this survey also revealed little provision of training in residency programs to support obesity management [28]. To our knowledge, this is the first study to survey family medicine residency leaders on the state of obesity education in their programs.

Despite the identified gaps in training related to management of patients with obesity during family medicine residency, the vast majority of program leaders in our study recognize the importance of providing obesity education and the challenges that arise with expanding such education. Graduate medical education has appropriately evolved to include and promote other topics relevant to primary patient care, such as opioid use and dependence [29]. Management of patients with obesity, however, still appears to be a relatively low priority. Although a lack of room in the curricula is reported as a major barrier by program leaders, the lower prioritization of obesity may also be due to other factors such as stigma against patients with obesity in the healthcare community [30].

Most programs are devoting considerable time to education about obesity. Rather than addressing the challenge of expanding time for obesity education, improvement may simply result from better aligning current training with the systematically developed OMEC competencies [18]. For example, less time could be devoted to understanding surgical procedures and more time to addressing stigma and its impact. One of the core initiatives of The Strategies to Overcome and Prevent (STOP) Obesity Alliance, a multi-organization collaboration dedicated to addressing policies, treatment, research, and education, is “Curating the Obesity Care Competencies” [31]. The goal of this STOP project is to support the implementation of the OMEC-developed obesity competencies by curating a collection of curricular material and tools for use by organizations to facilitate obesity education and training such as the Weight Can’t Wait Guide [32] as well as to develop a curricular case series highlighting successful competency integration strategies.

Our survey results suggest several opportunities for improving education about management of patients with obesity within existing structures. Lack of faculty with obesity expertise can be addressed by either leveraging existing multidisciplinary team members such as pharmacists to expand education on pharmacotherapy, as done in other chronic disease models, or using external resources, such as shared curricula from institutions already excelling in obesity education or widely available web-based CME (Continuing Medical Education) training sessions. Examples of CME include those developed by The Endocrine Society [33, 34], the Obesity Medicine Association [35] the Obesity Society [36] and the American Association of Clinical Endocrinology (AACE) [37]. Other available resources include online resources and webinars such as those offered by UConn Rudd Center for Food Policy & Obesity [38], the AACE Nutrition and Obesity Resource Center [39], and The Obesity Medicine Association [40, 41], Additionally, interactive training sessions, such as those developed by the American Heart Association [42], can be incorporated during clinical rotations to provide residents with specific skills that can be applied within busy, ambulatory care settings in which most will practice as primary care providers. Opportunities for training include patient-centric communication, counseling skills, and strategies to assess readiness and self-efficacy for behavior change [43]. Additionally, teaching integration of clinical and community partners could facilitate patient compliance and could similarly leverage the high prevalence of multidisciplinary members such as social work and nursing. Ultimately, development and endorsement of obesity education for family medicine residency programs by AAFP and STFM could have the greatest impact on the implementation of consistent training related to the treatment and management of obesity.

As our study is a baseline assessment, it could be repeated in the future once programs have been able to incorporate the OMEC competencies, in order to assess the progress of education and the impact of increased training on obesity management in U.S. family medicine residency programs. Initial examination of the impact of incorporating the OMEC competencies suggests that such incorporation will be well received. A recent study of a Midwest family medicine program assessing a new half-day teaching session based on the competencies demonstrated a positive impact on residents’ approach in managing obesity with more than 80% of residents surveyed after the session reporting that the content at least moderately impacted how they approached obesity management; comfort in working with patients with obesity and perception of their own biases improved significantly from baseline to immediately after the intervention and were sustained 15 months later [44].

### Limitations

The study has several limitations including a small sample size, as only 16% of family medicine residency programs responded to the survey, despite attempts to maximize response rates utilizing a variety of methods. This could be due to competing with the many other demands on residency program leaders’ time, or it could be indicative of low interest in the topic of obesity education. The sample size limited the number of subgroup analyses that could be conducted. Additionally, participation among certain groups was absent or limited, including military family medicine residency programs and programs located in New England, the Pacific Northwest, Appalachia, and parts of the South. Although these limitations may constrain generalizability of the results, the sample distribution served by responding programs was similar to that of invited programs with respect to types of institutions with which the programs are affiliated, populations served, and U.S. geographic region. These facts may support generalizability in this context.

Respondents appeared to be candid in their survey responses. Responder bias would likely have resulted in a larger percentage of programs which are more adequately addressing obesity in their curricula, or, conversely, fewer programs reporting inadequately addressing education of the condition; instead, our data show significant room for improvement. We acknowledge that respondents’ assessment of their curricula is likely to be subjective. Results are nonetheless informative and relevant for understanding obesity education related challenges faced by family medicine residency programs. The questionnaire was developed by researchers with extensive experience in survey design and medical residency training even though the survey was not formally validated, and response and other biases are possible. Obesity education can take multiple forms including informal or self-directed learning; this type of learning was not assessed in our study.

## Conclusions

Despite being in a unique position to provide obesity care, most recently trained family medicine physicians are likely not adequately prepared to manage patients with obesity. Training related to prevention and management of patients with obesity appears suboptimal in a large portion of family medicine residency programs. Although most family medicine residency programs surveyed cover some elements of obesity education to an extent, in a wide variety of settings, most of the OMEC obesity-related competencies are not yet adequately addressed. Our survey results point to several opportunities for leveraging existing resources to incorporate the current OMEC competencies. Such opportunities can contribute to improving the quality of family medicine residency education in obesity.

## Supplementary Information

**Additional file 1.** Family Medicine Residency Curriculum Survey. Questions from survey conducted among family medicine residency directors.

## Data Availability

The datasets used and/or analyzed during the current study are available from the corresponding author on reasonable request.

## Abbreviations

U.S.: United States
OMEC: Obesity Medicine Education Collaborative
AAFP: American Academy of Family Physicians
STFM: Society of Teachers of Family Medicine
ACGME: Accreditation Council for Graduate Medical Education
SD: Standard deviation
STOP: Strategies to Overcome and Prevent Obesity
CME: Continuing Medical Education
AACE: American Association of Clinical Endocrinology
