# Supplementary material for: Benchmarking of provider competencies and current training for prevention and management of obesity among family medicine residency programs: a cross-sectional survey
Source: BMC Fam Pract. 2021 Jun 24;22:132. doi: 10.1186/s12875-021-01484-y (PMC8229273; doi:10.1186/s12875-021-01484-y)
Supplement: Supplementary file 1 — Additional file 1. Family Medicine Residency Curriculum Survey. Questions from survey conducted among family medicine residency directors. [file 12875_2021_1484_MOESM1_ESM.docx]

**Additional File 1. Family Medicine Residency Curriculum Survey**

**SECTION S: sample PRELOAD AND SCREENING QUESTIONS**

**ALL RESPONDENTS**

**P1 Program name from target list**

[PROGRAMMER NOTE]

IF A VALIDATED RESPONDENT FROM THE SAME INSTITUTION HAS ALREADY COMPLETED THE SURVEY, DISPLAY “Thank you for your interest in this survey. Unfortunately, someone from your institution has already participated in this survey and we are currently limiting responses to one per institution. You may receive survey invitations from us for future studies on relevant topics.”

**ALL RESPONDENTS**

**P2 State from target list**

**ALL RESPONDENTS**

**P3 Administrative categories from target list**

**ALL RESPONDENTS**

**P4 Geographic settings served from target list**

**ALL RESPONDENTS**

**S1** Thank you for your interest in this survey. We appreciate your willingness to participate in this important research on obesity medicine education.

Before participating, KJT Group requires you to review the following information:

- Academic researchers from Columbia University Mailman School of Public Health and the Bariatric and Metabolic Institute at the Cleveland Clinic, in collaboration with Novo Nordisk Inc., are conducting this survey to **better understand the state of obesity education in family medicine residency programs in the United States**.
- KJT Group is a **global market research company**.
- You have been invited to participate as you may be familiar with the family medicine residency program curriculum, including where and how obesity is taught.
- Your participation involves completing this survey.
- We expect, on average, it will take respondents like yourself **15 minutes** to complete this survey.
- Findings of this research project may be published in scientific journals or presented at medical meetings. All the results will be presented in aggregate form; data will never be presented in a way that identifies individual institutions.
- Your responses will be kept **strictly confidential** and will never be associated with your name.
- Your **participation is voluntary,** and you may choose to stop participating at any time (withdraw consent).

Do you consent to these terms and wish to continue with the following survey on obesity medicine education?

1. Yes [CONTINUE]
2. No [TERMINATE]

[IF AGREES TO DISCLOSURE STATEMENT (S1/1), ASK S2. ALL OTHERS TERMINATE.]

**ALL RESPONDENTS**

**S2** What is your role in the family medicine residency program?

1. Program director
2. Associate or co-program director
3. Assistant director
4. Chief resident
5. Other, please specify: [MANDATORY TEXT BOX]

**ALL RESPONDENTS**

# **S3** How familiar are you with the ACGME’s learning objectives and requirements for Family Medicine?

1. Extremely
2. Very
3. Somewhat
4. A little
5. Not at all

[IF AT LEAST A LITTLE FAMILIAR (S3/1-4); CONTINUE; ALL OTHERS TERMINATE.]

**ALL RESPONDENTS**

**S100 FINAL QUOTA QUESTION [n=100]**

- At least a little knowledgeable about family medicine residency curriculum

Not qualified  **[n=9999]**

**SECTION 200: Family Medicine RESIDENCY CURRICULUM ASSESSMENT**

**ALL QUALIFIED RESPONDENTS**

**Q200** You have qualified for the full survey. Thank you for your responses thus far. The remainder of this survey should take approximately 15 minutes to complete. As a reminder, your responses to this survey are critical to the success of this research in helping the sponsor to better understand obesity education in family medicine residency programs. Your responses will be kept strictly confidential and only reported in combination with other respondents’ data. In addition, you may be asked certain questions for quality control purposes.

Please click the forward arrow to continue.

**ALL QUALIFIED RESPONDENTS**

**Q202** How long have you been at your current institution?

[RANGE 0-50]

[_|_] years

**ALL QUALIFIED RESPONDENTS**

**Q203** How long have you been in your current role as it is related to the family medicine residency curriculum?

[RANGE 0-50]

[_|_] years

**ALL QUALIFIED RESPONDENTS**

**Q205** Do you have any involvement in writing or submitting questions for the family medicine board certification exam?

1. Yes
2. No

**ALL QUALIFIED RESPONDENTS**

# **Q206** Are you involved in teaching/training residents?

1. Yes
2. No

[DISPLAY Q208A AND Q208B ON SAME SCREEN]

**ALL QUALIFIED RESPONDENTS**

# **Q208A** Is teaching residents about prevention of obesity in patients an intentional (i.e., formal or organized) educational objective in your program?

1. Yes
2. No

**Q208B** Is teaching residents about management of patients with obesity an intentional educational objective in your program?

1. Yes
2. No

**OBESITY IS AN INTENTIONAL EDUCATIONAL OBJECTIVE (Q208A/1)**

# **Q209A** In which setting is teaching about prevention of obesity in patients an intentional educational objective in your program?

*Please select all that apply.*

[RANDOMZE]

1. During teaching in inpatient rotations
2. Dedicated seminars, lectures, or conferences
3. Precepting in outpatient rotations and continuity clinics
4. Other, please describe: [ANCHOR] [MANDATORY TEXT BOX]

**OBESITY IS AN INTENTIONAL EDUCATIONAL OBJECTIVE (Q208B/1)**

# **Q209B** In which setting is teaching about management of patients with obesity an intentional educational objective in your program?

*Please select all that apply.*

[RANDOMZE]

1. During teaching in inpatient rotations
2. Dedicated seminars, lectures, or conferences
3. Precepting in outpatient rotations and continuity clinics
4. Other, please describe: [ANCHOR] [MANDATORY TEXT BOX]

**ALL QUALIFIED RESPONDENTS**

**Q209C** Which resources does your program currently use?

*Please select all that apply.*

[RANDOMIZE] [MULTIPLE-SELECT]

1. MKSAP
2. Harrison’s Principles of Internal Medicine
3. Goldman-Cecil Medicine
4. Essential Evidence Plus
5. Up to Date
6. Cochrane
7. Hopkins modules
8. Yale modules
9. Other, please specify: [ANCHOR] [MANDATORY TEXT BOX]

**ALL QUALIFIED RESPONDENTS**

# **Q210A** Approximately how many sessions of formal teaching (e.g., lectures, review sessions, journal club) per week do you offer your residents?

[RANGE 0-100]

[_|_] sessions

**ALL QUALIFIED RESPONDENTS**

# **Q210B** Approximately how many of these formal teaching sessions -over the entire course of residency training- are specifically on obesity (either prevention of obesity or management of patients with obesity)?

[RANGE 0-100]

Obesity prevention: [_|_] sessions

[RANGE 0-100]

Management of obesity: [_|_] sessions

**ALL QUALIFIED RESPONDENTS**

# **Q211** Approximately what percentage of teaching that occurs during delivery of clinical care (i.e., precepting in an outpatient setting or inpatient rotations) are specifically on managing of patients with obesity?

[RANGE 0-100]

[_|_] %

**ALL QUALIFIED RESPONDENTS**

**Q212** To what extent are each of the following topics covered/treated during your family medicine residency program?

[DISPLAY AS CAROUSEL; ONE ROW PER SCREEN.]

[COLUMNS]

1. Great extent
2. Some extent
3. Very little extent
4. Not at all

[RANDOMIZE] [ROWS]

1. Physical activity components of obesity (e.g., intensity, duration, frequency)
2. Physiology of obesity or weight regulation ((e.g., hunger hormone (ghrelin) and satiety hormones (leptin, PYY, CPK, GLP-1, amylin, etc.))
3. Etiologic aspects of obesity (e.g., circadian rhythm disorders, genetic and hypothalamic obesity)
4. Behavioral components of obesity (e.g., behavior change theory, motivational interviewing)
5. Psychosocial components of obesity (e.g., binge eating, not anorexia nervosa or bulimia)
6. Nutritional aspects of obesity (e.g., caloric balance, macronutrients)
7. Weight stigma and discrimination
8. Assessment tools of the patient with obesity (e.g., body composition analysis, metabolic testing)
9. Physical examination skills specific to assessing patients with obesity (e.g., measuring waist circumference, detecting / recognizing acanthosis nigricans)
10. Pharmacologic treatment of obesity (e.g., phentermine, locaserin, liraglutide)
11. Surgical treatment of patients with obesity (e.g., sleeve gastrectomy, gastric bypass)
12. Population aspects of obesity (e.g., public health policy)

**ALL QUALIFIED RESPONDENTS**

**Q213** For each of the following topics, indicate the type of education/training that is received.

*Please select at least one type per row.*

[GRID] [MULTIPLE-SELECT]

[ONLY DISPLAY r1-12 FROM Q212 THAT ARE COVERED AT LEAST A VERY LITTLE EXTENT (Q212_c1-3)

[COLUMNS]

1. Inpatient rotations
2. Dedicated seminars, lectures, or conferences
3. Outpatient precepting

[RANDOMIZE] [ROWS]

1. Physical activity components of obesity (e.g., intensity, duration, frequency)
2. Physiology of obesity or weight regulation ((e.g., hunger hormone (ghrelin) and satiety hormones (leptin, PYY, CPK, GLP-1, amylin, etc.))
3. Etiologic aspects of obesity (e.g., circadian rhythm disorders, genetic and hypothalamic obesity)
4. Behavioral components of obesity (e.g., behavior change theory, motivational interviewing)
5. Psychosocial components of obesity (e.g., binge eating, not anorexia nervosa or bulimia)
6. Nutritional aspects of obesity (e.g., caloric balance, macronutrients)
7. Weight stigma and discrimination
8. Assessment tools of the patient with obesity (e.g., body composition analysis, metabolic testing)
9. Physical examination skills specific to patients with obesity (e.g., waist circumference, acanthosis nigricans)
10. Pharmacologic treatment of obesity (e.g., phentermine, locaserin, liraglutide)
11. Surgical treatment of patients with obesity (e.g., sleeve gastrectomy, gastric bypass)
12. Population aspects of obesity (e.g., public health policy)

**ALL QUALIFIED RESPONDENTS**

**Q213B** To what extent does your program teach an approach to obesity management that integrates clinical and community systems as partners?

1. Great extent
2. Some extent
3. Very little extent
4. Not at all

**ALL QUALIFIED RESPONDENTS**

**Q213C** To what extent does your program emphasize the use of patient-centered communication when working with patients with obesity (e.g., using people-first language, active listening, incorporating shared decision-making)?

1. Great extent
2. Some extent
3. Very little extent
4. Not at all

**ALL QUALIFIED RESPONDENTS**

**Q214** Are there formal non-surgical clinical rotation opportunities in obesity (e.g., weight management clinic) for residents?

1. Yes
2. No
3. I don’t know

**ALL QUALIFIED RESPONDENTS**

**Q215** Are there formal surgical clinical rotation opportunities in obesity (e.g., bariatric surgery clinic) for residents?

1. Yes
2. No
3. I don’t know

**ALL QUALIFIED RESPONDENTS**

**Q216** Does your institution perform bariatric surgery?

1. Yes
2. No
3. I don’t know

**ALL QUALIFIED RESPONDENTS**

**Q217A** What other disciplines/ health care professionals participate in your inpatient rounds (i.e., are part of the medical team rounds) and participate in outpatient clinic team conferences (e.g., meetings of the ‘medical home’ team)?

*Please select all that apply*

[RANDOMIZE]

1. Nursing
2. Nutrition
3. Physical Therapy
4. Social Work
5. Psychology
6. Other, please describe: [ANCHOR] [MANDATORY TEXT BOX]

**ALL QUALIFIED RESPONDENTS**

**Q217B** Does your family medicine residency program offer working with non-medical providers (e.g., dietitians, psychologists) who care for patients with obesity?

1. Yes
2. No
3. I don’t know

**ALL QUALIFIED RESPONDENTS**

**Q219** How prepared do you personally feel in making a diagnosis of obesity?

1. Very prepared
2. Fairly prepared
3. Somewhat prepared
4. Not at all prepared

**ALL QUALIFIED RESPONDENTS**

**Q220** How prepared do you personally feel giving nutritional advice on obesity management?

1. Very prepared
2. Fairly prepared
3. Somewhat prepared
4. Not at all prepared

**ALL QUALIFIED RESPONDENTS**

**Q221** How prepared do you personally feel giving physical activity advice on obesity management?

1. Very prepared
2. Fairly prepared
3. Somewhat prepared
4. Not at all prepared

**ALL QUALIFIED RESPONDENTS**

**Q222** How prepared do you personally feel prescribing pharmacotherapy for management of patients with obesity?

1. Very prepared
2. Fairly prepared
3. Somewhat prepared
4. Not at all prepared

**ALL QUALIFIED RESPONDENTS**

**Q218** How prepared do you think your residents are to provide medical care for patients with obesity upon completion of residency?

1. Very prepared
2. Fairly prepared
3. Somewhat prepared
4. Not at all prepared

**ALL QUALIFIED RESPONDENTS**

**Q223** Please rate the need for resident education on these topics based on importance from “not at all important” to “very important”.

[COLUMNS]

1. Very important
2. Fairly important
3. Somewhat important
4. Not at all important

[RANDOMIZE] [ROWS]

1. Patient-centered treatment of weight & goal setting
2. Bringing up the topic of weight
3. Time management
4. Coding and billing for an obesity encounter
5. Comfort with pharmacotherapy
6. Comfort with referrals for weight loss surgery
7. Discussing weight and well-being
8. Discussing the relationship between weight and comorbidities
9. Physician role with patients who are overweight or have obesity

**ALL QUALIFIED RESPONDENTS**

**Q224** Have there been discussions about incorporating or expanding formal education of obesity in your family medicine residency program?

1. Yes
2. No
3. I don’t know

**ALL QUALIFIED RESPONDENTS**

**Q225** To what extent is expanding education/training in obesity a priority for your residency program?

1. High priority
2. Medium priority
3. Low priority
4. Not a priority

**ACTIVELY DEVELOPING OBESITY CURRICULUM (Q224r1)**

**Q226** Which of the following best describes when you expect to implement or expand your obesity education curriculum?

1. Within the next year
2. 1-2 years from now
3. More than 2 years from now

**ALL QUALIFIED RESPONDENTS**

**Q227** For each the following factors, please indicate to what degree it acts as a barrier to integrating obesity education into your curriculum.

[GRID]

[COLUMNS]

1. Not a barrier
2. Small barrier
3. Moderate barrier
4. Large barrier

[RANDOMIZE] [ROWS]

1. Lack of resident interest
2. Lack of faculty interest
3. Lack of room (time) in the curriculum
4. Lack of faculty expertise
5. Financial

**ALL QUALIFIED RESPONDENTS**

**Q228** Obesity impacts care for many other health conditions. Should management of patients with obesity be taught:

1. As a separate discipline
2. Incorporated as portions of teaching done on management of other conditions
3. Both
4. Neither, please comment: [ANCHOR] [MANDATORY TEXT BOX]

| **Section 100: Demographics** |
| --- |

**ALL QUALIFIED RESPONDENTS**

**Q102** Is there a board-certified obesity medicine specialist (a physician who has a certification in obesity medicine from the American Board of Obesity Medicine) at your institution?

1. Yes
2. No
3. I don’t know

**ALL QUALIFIED RESPONDENTS**

**Q103** Is your facility a bariatric center of excellence?

1. Yes
2. No
3. I don’t know
